# ASSOCIATIONS BETWEEN DAILY STRESSORS AND ALCOHOL USE IN THE MIDUS STUDY

**DOI:** 10.1093/geroni/igad104.2514

**Published:** 2023-12-21

**Authors:** Sara Miller, David Almeida

**Affiliations:** Pennsylvania State University, State College, Pennsylvania, United States; The Pennsylvania State University, State College, Pennsylvania, United States

## Abstract

Daily stressors are minor but frequent challenges of everyday life (e.g., arguments). Although daily stressors are typically less severe than stressful life events, daily stressors can have more immediate effects on wellbeing. Despite this relevance, there is limited research evaluating the influence of daily stressors on alcohol use in older populations. This study examined daily associations between stressor characteristics (exposure, type, and severity) and alcohol use and evaluated differences in associations for younger/middle-aged (< 65 years) vs older adults (65+ years). Participants (n=1,603; Mage=52.82) were non-abstaining adults from the Midlife in the United States (MIDUS) study and the MIDUS Refresher who participated in 8-day diary projects. Survey items included stressor exposure (1=yes), stressor types (interpersonal, work, home, network), stressor severity (0=not at all; 1=not very; 2=somewhat; 3=very), and number of drinks. Covariates included the day of the week, sociodemographic characteristics, personality traits, psychological factors, and health behaviors. Multilevel linear regression analysis revealed that neither daily stressor exposure nor stressor severity was associated with reported alcohol use (p>0.05), but there were differences in alcohol use by stressor type. Specifically, days with interpersonal stressors were associated with reporting more drinks (p=.049), while days with work stressors were associated with reporting fewer drinks (p=.001). Further, age significantly moderated the relationship between any stressor exposure and reported alcohol use (p=.035), such that stronger relationships were observed for older adults than younger/middle-aged adults. Study findings highlight the importance of considering stressor characteristics when evaluating the covariation of daily stressors and alcohol use.

